# mmView: a web-based viewer of the mmCIF format

**DOI:** 10.1186/1756-0500-4-121

**Published:** 2011-04-12

**Authors:** Petr Čech, Daniel Svozil

**Affiliations:** 1 Department of Computing and Control Engineering, Faculty of Chemical Engineering, Institute of Chemical Technology, Technická 5, CZ-166 28 Prague, Czech Republic; 2 Laboratory of Informatics and Chemistry, Faculty of Chemical Technology, Institute of Chemical Technology, Technická 5, CZ-166 28 Prague, Czech Republic

## Abstract

**Background:**

Structural biomolecular data are commonly stored in the PDB format. The PDB format is widely supported by software vendors because of its simplicity and readability. However, the PDB format cannot fully address many informatics challenges related to the growing amount of structural data. To overcome the limitations of the PDB format, a new textual format mmCIF was released in June 1997 in its version 1.0. mmCIF provides extra information which has the advantage of being in a computer readable form. However, this advantage becomes a disadvantage if a human must read and understand the stored data. While software tools exist to help to prepare mmCIF files, the number of available systems simplifying the comprehension and interpretation of the mmCIF files is limited.

**Findings:**

In this paper we present mmView - a cross-platform web-based application that allows to explore comfortably the structural data of biomacromolecules stored in the mmCIF format. The mmCIF categories can be easily browsed in a tree-like structure, and the corresponding data are presented in a well arranged tabular form. The application also allows to display and investigate biomolecular structures via an integrated Java application Jmol.

**Conclusions:**

The mmView software system is primarily intended for educational purposes, but it can also serve as a useful research tool. The mmView application is offered in two flavors: as an open-source stand-alone application (available from http://sourceforge.net/projects/mmview) that can be installed on the user's computer, and as a publicly available web server.

## Background

The Protein Data Bank (PDB) [1] is a publicly available central repository containing experimentally determined structures of proteins, nucleic acids and complex assemblies. The core of the system are relational databases, together with the so-called „PDB archive“ - a collection of the manually curated flat (i.e. ASCII) files. They are available for download in three different formats: the legacy PDB format [2-4], the mmCIF format [5,6] and the PDBML format [7].

The PDB format [8] is still the most widely supported and used because of its simplicity. It uses fixed format records (i.e. the individual entries must be put in specified character positions) with maximum line's width of 80 characters (a reminiscence of Fortran's 80 column wide punched cards), and allows for description of atomic coordinates, chemical and biochemical features, experimental details of structure determination, and some structural features such as secondary structure assignments or biological assemblies. The current PDB format, being a legacy format, cannot fully address many informatics challenges related to the growing amount of structural data. The main limitation is the fixed width format placing absolute limits on the size of data items. For instance, the maximum number of atoms represented in a single PDB file is limited to 99 999, and large molecular systems, such as ribosomal units, cannot be represented in a single PDB entry. The experimental details are stored in REMARK records that are relatively easy for a human to read, however the automatic extraction of information from these records is rather difficult. PDB format also suffers from serious internal inconsistencies, such as relation between the sequence defined by the SEQRES records and the sequence derived from the observed residues within the ATOM records.

To overcome the limitations of the PDB format, a new textual format mmCIF was released in June 1997 [5,6]. mmCIF is the extension of the Crystallographic Information File (CIF) [9] format developed by the International Union of Crystallography (IUCr) and used for description of small molecule structures and associated difraction experiments. The format of the CIF dictionary (and the data based on that dictionary) conforms to a restricted version of the Self Defining Text Archive and Retrieval (STAR) representation [10]. STAR is a general ontology framework defining a set of encoding rules. These rules are then used by the Dictionary Definition Language (DDL) that enables the definition of various terms needed by a given discipline. The DDL provides a convention for naming and defining data items within the dictionary, declaring specific attributes of those data items, and for declaring relationships between data items. The STAR encoding rules and the DDL are widely used to develop a variety of domain specific dictionaries [11], e. g. the powder diffraction dictionary [12] or an NMR dictionary [13], including the Crystallographic Information File (CIF). The CIF dictionary was extended to include data relevant to the macromolecular experiments, and the mmCIF (macromolecular CIF) was created. The version 1.0 of the mmCIF format was further expanded by more than 100 new definitions leading to the release of the mmCIF 2.0 in the Fall of 2000.

**Figure 1:**
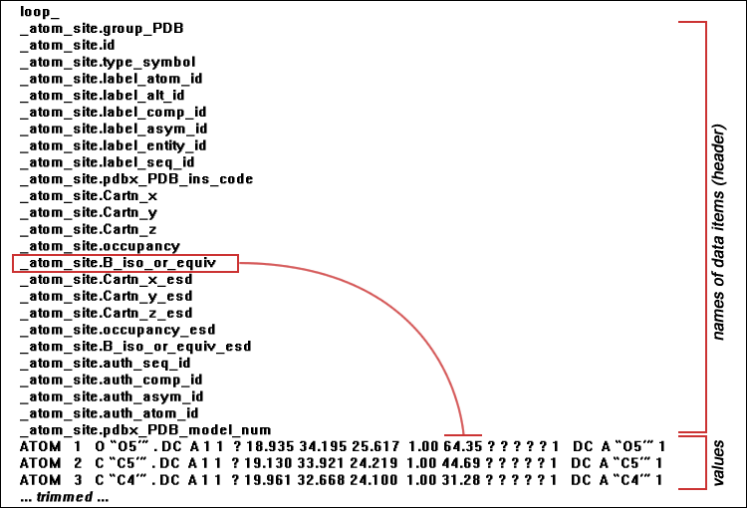
**The basic principles of the mmCIF syntax**.

An mmCIF file consists of a series of *name-value *pairs (a *data item*) defined by STAR, where the data name is preceded by a leading underscore (_). The *name *matches the entry in the mmCIF dictionary in which the characteristics of that data item are explicitly defined. Where multiple values for the same data item exist, the name of the data item is declared in a header and the associated values follow in rotation defined by a *loop_ *construct (only a single level of *loop_ *is allowed). The basic principles of the mmCIF syntax are illustrated in the trimmed representation of atomic coordinates (Figure 1).

The *name *construct is of the form *_category.extension *(e.g. _atom_site.group_PDB) where category defines a natural grouping of data items contained within a single *loop_*. The values corresponding to the names from the header are given in individual lines (corresponding to individual data items). Thus, e.g. the value of the isotropic atomic displacement parameter (given by _atom_site.B_iso_or_equiv name) is the 15^th ^value in each of the lines (Figure 1). Unknown data values are represented by a question mark (?), and undefined data are represented by a period (.) (Figure 1).

Compared to PDB, mmCIF provides extra information which has the advantage of being in a computer readable form. However, the biggest advantage of the mmCIF - well-formed, consistent (though also not perfect [14]) and computer readable format for storing the macromolecular structural data - becomes its disadvantage if a human must read and understand the stored data, as demonstrated in the example above (Figure 1). This makes difficult not only the adoption of mmCIF files, but also lays barriers in the educational process, e.g. in classes of structural biology or structural bioinformatics.

The importance of the mmCIF format is demonstrated by the fact, that it represents the data standard upon which the PDB is built [1]. PDB uses the data processing tool MAXIT, an integrated system helping to ensure that the data submitted are consistent with the mmCIF dictionary. In addition the schema of the PDB's core database is a subset of the conceptual schema specified by the mmCIF dictionary. Thus, the knowledge of the mmCIF format becomes essential for each scientist dealing with biomacromolecular structures. While software tools exist to help to prepare mmCIF files [15], the number of available systems simplifying the comprehension and interpretation of the mmCIF files is limited [16]. Therefore a web-based application mmView allowing for comfortable exploring of the biomolecular structural data stored in the mmCIF format was developed.

## Implementation

**Figure 2:**
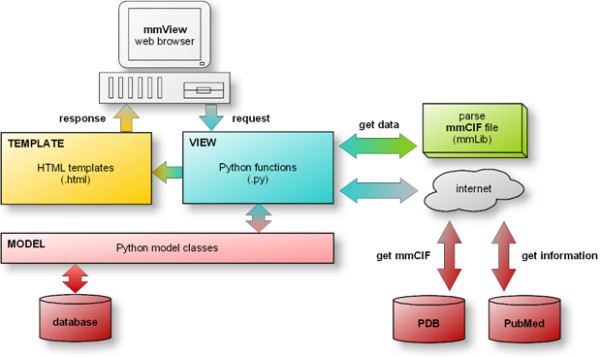
**mmView software architecture**. The mmView web application uses Model-Template-View (MTV) software architecture. The MTV architecture provides a way to separate the web interface (template layer) from the domain logic (model and view layers) making it easier to implement and modify each of these components independently. The View layer receives the request from the user's browser, invokes a desired functional operation and returns the template (i.e. the graphical data representation) as a response to the browser.

The mmView application is implemented as a dynamic web system using open source scripting language Python [17,18]. Python, which popularity is steadily rising among the bioinformatics community [19], is a modern object-oriented programming language that is easy to learn, easy to read, multiplatform and offers a wealth of available ready to use modules, as well as a strong support for integration with other languages and tools. To ease the development of the mmView system, a Python-based web framework Django [20,21] was utilized. Django is a high-level web framework based on a model-template-view (MTV) software architectural pattern (Figure 2), which represents a flavor of widely accepted model-view-controller (MVC) architecture. The MVC architecture provides a way to separate the user interface from the domain-specific representation of the data (e.g. data stored in the relational database), and from the domain logic (i.e. from functional algorithms that handle information exchange between a database and a user interface). The separation of the application in the three layers makes it easier to implement and modify each component independently. The Django's architecture assures portability to the user's preferred operating system (Linux, MS Windows, Mac OS X), web server (Apache) and database server (SQLite, MySQL, PostgreSQL). The core of the Django's MTV architecture consists of three layers. The Model layer defines underlying data storage mechanism, and provides easy access to the available data. The View defines which data will be presented to the user (including also the inner programmatic processing of the data), and the Template layer is responsible for the displaying of this data.

The mmView application is available in two flavors: as a standalone application that can be downloaded from http://sourceforge.net/projects/mmview and installed locally on user's computer, and as the ready-to-use online version available at http://ich.vscht.cz/~cechp/mmcif. To simplify the installation of the standalone version, the SQLite3 database (version 3.6.10) [22], which does not require a separate server to run and can be easily configured, is used. While SQLite3 provides an excellent development alternative for applications that are predominantly read-only or require a smaller installation footprint, it is not suitable for deployment in the multi-user environment. Thus the online version of mmView uses MySQL database engine (version 5.0.51a) [23]. However, the change of database backend is the matter of one directive in Djangos' configuration files, and user can therefore switch between various database engines easily. To make the application to have a look-and-feel of a desktop application Asynchronous JavaScript and XML (AJAX) is used for concurrent updating of parts of the web-site without the need of full page refreshes. The AJAX functionality is implemented utilizing the jQuery [24] library.

The View layer (Figure 2) controls access to the web services called from the mmView system. This includes the PDB server, from which mmCIF files are downloaded, and the PubMed service [25], from which additional bibliographic information is received. Several third party specific-purpose modules are employed in the mmView system. The key component is the mmLib [16] parser - a library for the analysis and manipulation of macromolecular structural models stored in mmCIF format. It significantly simplifies programmatic access to the biomolecular structures by parsing mmCIF (or PDB) files directly into the set of Python objects (Figure 2). To display structures in 3D the open-source software molecular structure viewer Jmol [26] is integrated into the mmView application, and its look and feel is adjusted usings Jmol scripting abilities.

## Results and discussion

**Figure 3:**
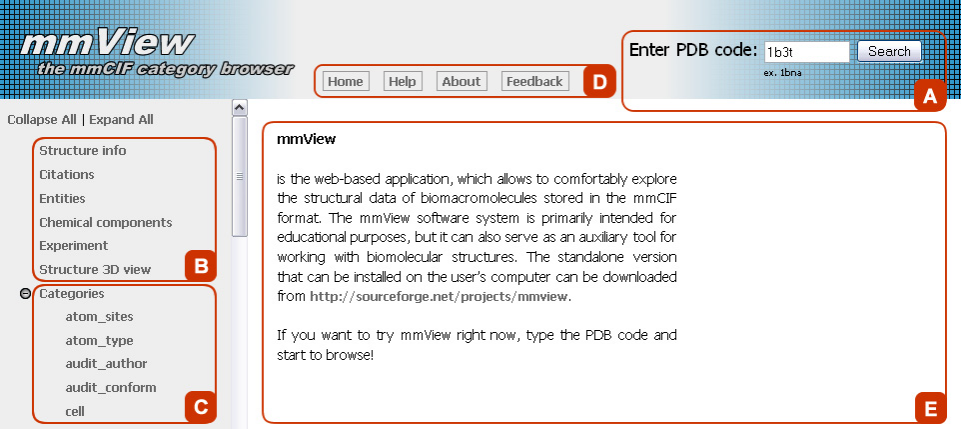
**Homepage of the mmView application**. a) Form field for inserting structure's PDB code. b) Aggregated view combines the key information obtained from various categories in the mmCIF file and from external sources. c) Category view lists all categories available in the mmCIF file. d) The main menu of the mmView application. e) The main viewing area.

The workflow of the mmView application begins by typing a PDB code to the form field placed at the top of the mmView application (Figure 3a). mmView stores once accessed structures locally at the servers' cache, which is searched first. The structure is downloaded (in mmCIF format) from the PDB database only if it is not found in the cache. The mmCIF file is then parsed by the mmLib module, and the leftside menu is generated (Figure 3b and 3c). The integrated help is available from the homepage header (Figure 3d), and is thus visible irrespective of the content in the main viewing area (Figure 3e).

All information about structure is presented in two different ways. The leftside menu is divided in two parts, where the first part (Figure 3b, further referred to as Aggregated view) represents the most important information about the structure. The second part (Figure 3c, further referred to as Category view) contains list of all categories presented in the analyzed mmCIF file.

### Aggregated view

The Aggregated view contains six topics covering various aspects of molecular structure. Individual topics, containing data combined from different mmCIF categories, simplify the access to the most significant information. Hypertext links to the structure's original mmCIF categories are available at every Aggregated view page. In Aggregated view the standard data present in mmCIF file may also be enriched by the additional information obtained from external resources. Each of the topics is described in the integrated help. Aggregated view contains the following topics.

#### Structure info

Basic information about the investigated structure is summarized under the Structure info topic and contains the PDB ID, the full title of the primary reference, names of authors and dates of deposition and release of the structure. If the cif file contains sections describing the presence of mutations, ligands, modified residues or oligomeric state of the structure, such information is also displayed in this section.

#### Citations

**Figure 4:**
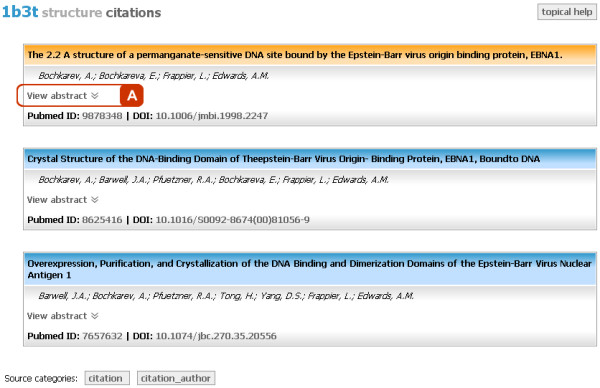
**Aggregated view - Citations**. Topic showing literature citations referring to the given structure. a) Abstract of the citation is available if present on PubMed, and is displayed upon clicking the „View abstract“ link.

Citations topic (Figure 4) displays the information about the publications relevant to the given structure. It includes not only titles and authors of papers present in the mmCIF file, but also publications' Digital Object Identifiers (DOI), PubMed IDs and their abstracts (Figure 4a). These supplementary data are acquired from bibliographic database PubMed using its Application Programming Interface (API). The mmView sends an HTTP request consisting of the article's title and authors' names to the PubMed's API, which responds back by sending the requested data in XML format. XML data are then parsed using the Simple Application interface for XML (SAX) [27], and their content is displayed on the web page.

#### Entities

Biomolecular structure consists of one or more entities, large units describing the chemistry of molecules under investigation. Entities are of three types: polymer (DNA, RNA or protein), non-polymer (e.g. ions), and water. Entities consist of chemical components - all monomers (residues, ions, water) found in the structure. Entities are summarized in the Entities topic, and components are displayed in the Chemical components topic (for its description, see next paragraph).

The Entities topic gives general overview about all entities found in the structure. If the entity is of the polymer type, short description and sequence using one letter codes (codes closed in brackets correspond to non-standard residues) is displayed. Instead of original mmCIF terminology used in *_entity_poly.type *item (polypeptide D/L, polydeoxyribonucleotide, polyribonucleotide, polysacharide D/L) entity types are labeled as protein, DNA, RNA or polysacharide. For all other entities (non-polymer, water) code and description is displayed. The image of the chemical structure will appear on roll over the non-polymer entity code.

#### Chemical components

**Figure 5:**
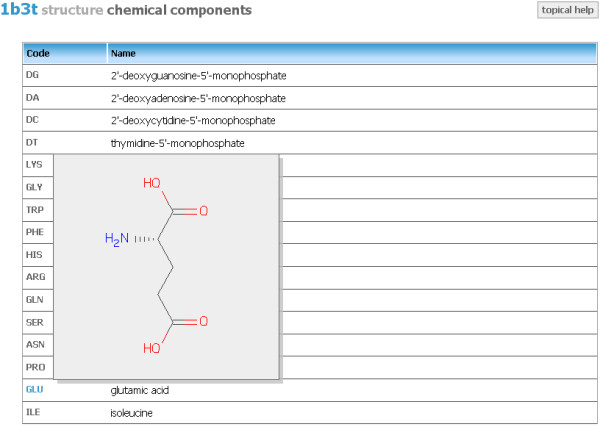
**Aggregated view - Chemical components**. Topic displaying information about all monomer units present in the inspected structure. The chemical structure of the compnent is showed upon rolling over its code.

The Chemical components topic (Figure 5) displays all monomer units biomolecular structure consists of. The table of chemical components contains standard two-letter (for nucleic acids residues) or three-letter (for protein residues) codes together with their chemical names. If rolling with the mouse cursor over the units' code the image with the chemical structure of the given component pops up (Figure 5).

#### Experiment

Experimental details and conditions are summarized in the Experiment topic. The provided information depends on the type of experimental method. Two most common experimental approaches for biomolecular structure determination are X-ray crystallography, and nuclear magnetic resonance (NMR).

**Figure 6:**
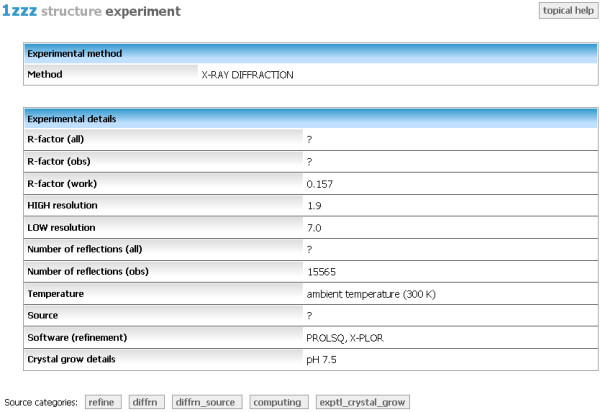
**Aggregated view - Experiment**. Topic displaying experimental details of X-ray diffraction method.

For X-ray diffraction experiments the following attributes, if given in mmCIF, are displayed (Figure 6):

• residual factors R for all reflections that satisfy the given resolution limits

• high and low resolution of interplanar spacings for the reflection data used in the refinement

• number of all and observed reflections

• ambient temperature

• source of diffraction rays

• crystal grow details

• the pH at which the crystal was grown

• the pH of the solution

• software used for refinement

For NMR experiments the following attributes, if given in mmCIF, are displayed:

• strength of the magnetic field

• the pH at which the NMR data were collected

• number of models

• software used in structure modelling

#### Structure 3D view

**Figure 7:**
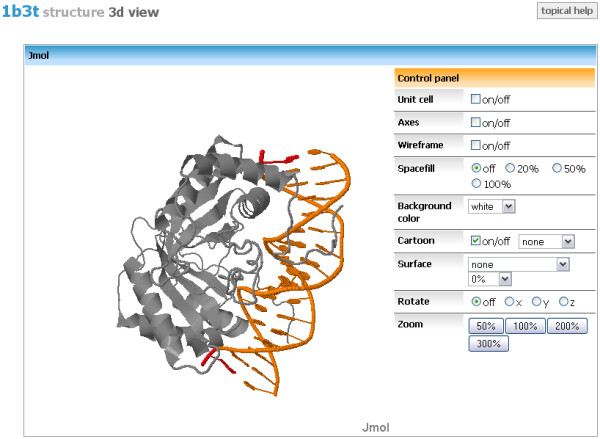
**Aggregated view - Structure 3D view**. Implemented Jmol application allows to view and investigate the structure in 3D form.

The last topic of Aggregated view offers complex survey of the structure in 3D form (Figure 7) utilizing the Jmol application [26]. Jmol provides plenty of possibilities to explore the whole biomolecular structure in many various ways. Whole Jmol's functionality is available through the menu appearing on the right mouse click. In addition, Jmol also offers the possibility to script its user interface, and to embed all its features directly to the HTML code. This capability is utilized in mmView application to create a customized 3D visualization environment. The following elements are easily accessible from the main application window (Figure 7):

• show/hide unit cell

• show/hide axes

• show/hide wireframe model of the structure

• change size of the spacefill of atoms

• change the background color

• view the structure in a cartoon representation

• explore the solvent accesible and Van der Waals surfaces

• rotate the structure

• zoom the structure

### Category view

The expandable Category view (Figure 3c) lists all categories available in the mmCIF file. Each item represents the original table stored in the mmCIF file without presenting any additional data (e.g. citation information from the PubMed database). Only the categories present in the given mmCIF file are shown. The category help available for each category is distinguished from the mmView's integrated help by using the icon with circled question mark. This depiction indicates that link leads to external resources, in this case to the HTML version of the IUCr standard mmCIF dictionary. In this way the up-to-date information is always available for users of the the mmView system.

## Disadvantages

mmView can display only the information that is available in the corresponding mmCIF file. However, if the data in the mmCIF file are not correct or the file contains errors, these are shown as they are. The number of requests that can be made on the PubMed server is currently limited to three requests every second (state of 19. 7. 2010). Thus, depending on the number of publications, generating the Citations topic can be slow. The dynamically generated leftside menu contains only categories present in the given mmCIF file. However, parsing large mmCIF files is a slow procedure, and thus it can produce additional delays in displaying leftside menu, especially for large structures. Another drawback of the mmView application is the fact, that at present it does not allow to investigate custom mmCIF files. Only structures from the PDB database can be studied, and their PDB IDs must be known in advance utilizing e.g. the search capability of the PDB web site.

## Future development

Future development directly relates to the above mentioned disadvantages of the mmView application. The delays can be avoided by deploying the relational database for storing critical data such as publication abstracts or lists of categories and topics of individual structures. The incorporation of simple form allowing to search PDB database directly from mmView, as well as the possibility to upload custom users' mmCIF files, are also planned for the next version of the application.

## Conclusions

The mmView application provides a simple but powerful tool for researchers in the fields of tructural biology, and structural bioinformatics. mmView is well suited as an educational tool (it is succesfully used by authors in their course of Structural bioinformatics), but can also serve as a research tool for exploring the details about biomolecular structures. Its online version does not require any installation and provides an intuitive and easy-to-use interface. In addition, the version that can be installed localy on the end-user's computer is also available.

## Availability and requirements

• **Project name: **mmView

• **Project home page: **http://ich.vscht.cz/~cechp/mmcif*(ready-to-use online version)*, http://sourceforge.net/projects/mmview*(stand-alone application)*

• **Operating system**: Platform independent, any modern web browser needed

• **Programming language**: Python

• **Other requirements**: Python 2.5.4, Django 1.0, NumPy 1.2.1, PymmLib 1.0, JRE 1.6

• **License: **GNU General Public License (Version 2)

• **Any restrictions to use by non-academics: **no restrictions

## Competing interests

The authors declare that they have no competing interests.

## Authors' contributions

PČ designed and implemented the software and drafted the manuscript. DS instigated the study, participated in its design and coordination, and helped to draft the manuscript. Both authors read and approved the final manuscript.
